# Relationship between preoperative lymphocyte to monocyte ratio and surgery outcome in type A aortic dissection

**DOI:** 10.3389/fsurg.2022.1103290

**Published:** 2023-01-05

**Authors:** Ming Ma, Feng Zhu, Fudong Fan, Jun Pan, Hailong Cao, Qing Zhou, Dongjin Wang

**Affiliations:** ^1^ Nanjing Drum Tower Hospital Clinical College of Nanjing Medical University, Nanjing, China; ^2^ Department of Thoracic and Cardiovascular Surgery, the Affiliated Drum Tower Hospital of Nanjing University Medical School, Nanjing, China; ^3^ Institute of Cardiothoracic Vascular Disease, Nanjing University, Nanjing, China

**Keywords:** aortic dissection, surgery outcome, lymphocyte, monocyte, preoperative

## Abstract

**Background:**

Lymphocyte-to-monocyte ratio (LMR) is associated with the mortality of cardiovascular diseases. However, the relationship between preoperative LMR and the prognosis of patients with Stanford type A aortic dissection (TAAD) undergoing surgical treatment remains to be determined.

**Methods:**

We enrolled 879 patients with TAAD undergoing surgical treatment between January 2018 and December 2021. Patients were divided into two groups: the Deceased group and the Survived group. The baseline clinical and operative characteristics of the two groups were compared and analyzed.

**Results:**

In univariate and multivariate logistic regression analysis, the association between LMR and in-hospital mortality was significant, and LMR (OR = 1.598, 95% CI 1.114–2.485, *P* = 0.022) remained significant after adjusting for confounders.

**Conclusion:**

Lower LMR may be independently associated with higher in-hospital mortality in TAAD undergoing surgical treatment.

## Introduction

Aortic dissection (AD) is a serious cardiovascular disaster, which is caused by a lesion in the intima of the aortic wall and leads to high-velocity blood flow entering the middle layer of the artery, and gradually extending and stripping the intima and tunica media of the aorta forming a true and false lumen. Half of the patients died within 48 h after symptoms onset without immediate appropriate medical treatment (1). In clinical practice, AD can be classified into Stanford type A AD (TAAD) when the ascending aorta is involved and Stanford type B AD (TBAD) when the incision of the aortic lesion is located in the descending segment of the aorta. TAAD accounted for 75% of cases with high mortality reaching 90% if untreated in time, which makes it the most common and lethal type of AD (2).

It is generally believed that hereditary tissue connective disease (ITCD), hypertension, and atherosclerosis (ATS) are the main causes related to AD (3). In recent years, more and more *in vivo* and *in vitro* research have shown immune inflammatory reaction may play key roles in the development of AD and is related to poor prognosis of patients (4). White blood cell subtypes, monocyte counts, and neutrophil-to-lymphocyte ratio (NLR) have been assessed as prognostic biomarkers for various cardiovascular diseases (5, 6).

Lymphocyte-to-monocyte ratio (LMR), as a new marker of the systemic inflammatory response, is widely used to predict the prognosis of cancer patients (7). In recent years, LMR has also been found a potential value associated with the mortality of cardiovascular disease (CVD) in the general population and hemodialysis patients (8). However, different centers had different findings between preoperative inflammatory response with the prognosis of AD in different treatment strategies (9, 10). Therefore, as the regional center for the aortic disease of Jiangsu Province with an official certificate, we conducted this observational study focusing on patients with TAAD who underwent surgery to assess the association between the admission LMR and in-hospital prognosis.

## Materials and methods

### Population and setting

In this single-center retrospective cohort study, we recruited 1,089 patients with TAAD who underwent surgical treatment at the Nanjing Drum Tower Hospital Clinical College of Nanjing Medical University (Nanjing, China) between January 2018 and December 2021. Patients were divided into the Deceased group and the Survived group. All TAAD patients were diagnosed by multidetector computed tomographic angiography. The exclusion criteria were as follows: the time of onset was more than 14 days or unknown; drugs that affect blood cell counts such as antibiotics and glucocorticoids have been used; acute and chronic hepatic and renal insufficiency unrelated to TAAD; tumor; active infections or autoimmune diseases; incomplete information; traumatic TAAD.

The study was approved by the institutional review board of Nanjing Drum Tower Hospital (2020-185-01). Written informed consent for participation in this study was waived due to the retrospective, noninterventional study design. All patient data were kept confidential.

### Data collection

Clinical information of patients was obtained through review of medical records in strict accordance with the inclusion criteria. The patient's venous blood samples, which were used for pre-op examination, were obtained within 1 h after admission to the cardiovascular ICU, and complete blood count was measured by the Department of Laboratory in the Nanjing Drum Tower Hospital, including white blood cell counts and types, lymphocyte counts and types, monocyte counts and other routine tests. The LMR was calculated by dividing the absolute lymphocyte counts by the absolute monocyte counts. Also, we collected all the demographic profiles, including age, gender, medical history, smoking and alcoholic usage. Cardiopulmonary bypass time (CPB time), mechanical ventilation hours and ICU stay time was also measured. The end point of the study was defined as all-cause mortality during hospitalization.

### Definitions

All TAAD subjects had image information from the chest or abdominal computed tomography (CT). AD diagnosis was conducted *via* Computed Tomography Angiography (CTA) (The 2014 European Society of Cardiology Guidelines for the Diagnosis and Treatment of Aortic Diseases). The time of treatment after symptoms onset is closely related to the prognosis of AD. Based on our AD database, we analyzed the expression of inflammatory cells in TAAD patients at hyperacute and acute stages; chest pain or other related symptoms occur no more than 14 days before admission. We use the definition of “prolonged CPB time” in the study of Roh (11) and Qiu (12) was “CPB time >180 min” for subgroup analysis. According to the KDIGO guideline (13), we choose the following: (1) small changes in serum creatinine (≥0.3 mg/dl or 26.5 mmol/L) when they occurred within 48 h; (2) a maximal change in serum creatinine ≥1.5 times the baseline value until postoperative day 7 compared with preoperative baseline values. The patients were subsequently categorized according to their highest levels of post-operative serum creatinine.

### Statistical analysis

Gaussian numerical variables are expressed as mean ± standard deviation, while other variables are presented as median (25–75th percentile), and categorical variables as number (percentage). Data were compared by the Student's *t*-test, Mann–Whitney *U* test, or *χ*^2^ test as appropriate. All *P* values are two-tailed. The alpha level was set as 0.05. R software (version 4.1.3) was used for data analysis. R packages “tableone” and “ggplot2” were used for basic statistics and tables. The multivariate logistic regression analysis was performed to explore the relationship between LMR levels and mortality in patients with TAAD. To further analyze the relationship between LMR and death in TAAD, LMR was transformed from a continuous variable to a categorical variable (through trisection of LMR concentrations). Odds ratios (ORs) and 95% CI were calculated.

## Results

**Figure 1:**
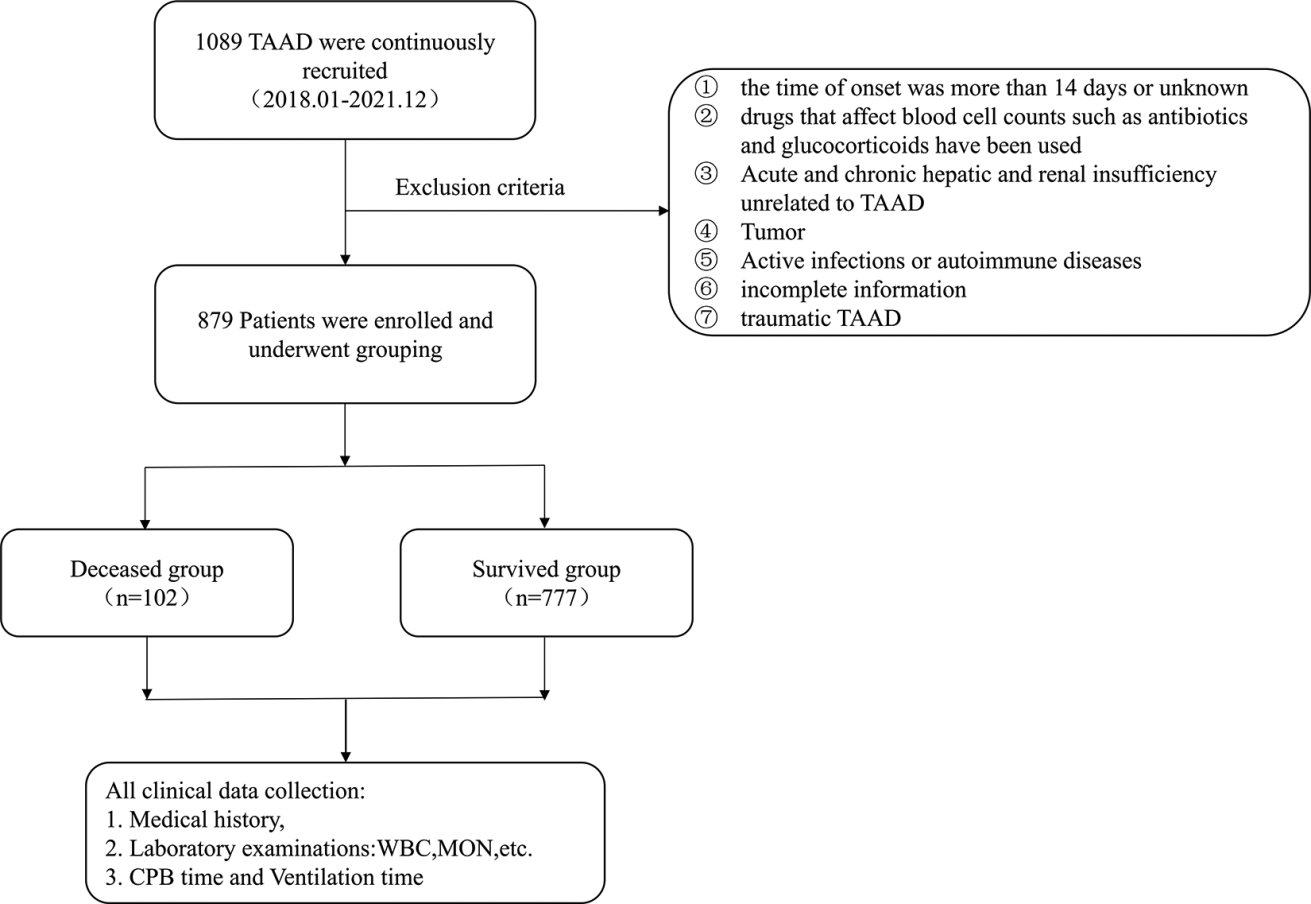
The flowchart of the selected population in this study.

From January 2018 and December 2021, a total of 1,089 TAAD were enrolled. We excluded 210 patients who met the exclusion criteria, then other 879 TAAD subjects were analyzed as two groups, of whom 777 survived and 102 deceased. The overall study design is shown in Figure 1. All patients in this study underwent CPB surgery. A total of 737 patients were at the hyperacute stage (symptoms onset less than 24 h on admission), while other 142 patients were at the acute stage. 23 patients had a history of cardiac surgery. 162 patients underwent the Bentall procedure, 29 patients had the Wheat technique and 8 patients had the David procedure during the surgery. There were 157 TAAD patients (17.9%) who had total aortic arch replacement. All subjects had deep hypothermic circulatory arrest (DHCP) during TAAD repair.

### Baseline clinical and operative characteristics

The baseline characteristics and operative details of the study population are shown in Table 1. The patients of the Deceased group were older at 56 years (median, IQR: 49–67) than others at 54 years (median, IQR: 44–64) (*P* = 0.009). There was no significant difference in gender proportion, BMI, smoker, drinker, hypertension, or diabetes mellitus (*P* > 0.05). While the difference in white blood cell count, neutrophil count, aspartate aminotransferase, serum creatinine, monocyte count, D-dimer, fibrinogen, and LMR among the two groups were statistically significant (*P* < 0.05). The CPB time of the two groups was 236 min (median, 189.0–312.5) and 198 min (median, 168.0–239.0), respectively, the difference in prolonged CPB time was statistically significant (*P* < 0.001). After surgery, the patients in the Deceased group had a longer ventilation time of 60 h (median, 21.0–148.0) than in the Survived group of 19 h (median, 12.0–52.0) (*P* < 0.001). While the postoperative AKI and length of ICU stay were no significant differences between the two groups.

**Table 1:** Baseline clinical and operative characteristics of study population.

Characteristic	Deceased *N* = 102	Survived *N* = 777	*P* ^a^
Female, *n* (%)	28 (27)	179 (23)	0.32
Age, years	56 (49.0 67.0)	54 (44.0 64.0)	**0.009**
Body mass index, kg/m^2^	25.2 (23.1 27.7)	25.6 (23.0 28.10)	0.26
Smoking, *n* (%)	31 (30)	196 (25)	0.26
Drinking habit, *n* (%)	24 (24)	144 (19)	0.23
Hypertension, *n* (%)	77 (75)	524 (67)	0.10
Diabetes mellitus, *n* (%)	4 (3.9)	29 (3.7)	0.79
Time to onset, *n* (%)			**0.032**
Hyperacute	93/102 (91)	644/777 (83)	
Acute	9/102 (8.8)	133/777 (17)	
Prolonged CPB time	83 (81)	505 (65)	**<0.001**
Ventilation time, h	60 (21.0 148.0)	19 (12.0 52.0)	**<0.001**
Postoperative AKI	13 (13)	60 (7.7)	0.084
ICU stay, days	6 (3.0 12.0)	4 (3.0 7.0)	0.130
**Laboratory parameters**			
White blood cell count, 10*9/L	12.75 (10.50 16.05)	12.10 (9.40 14.90)	**0.026**
Neutrophil percentage, *n* (%)	85.95 (81.65 89.70)	86.60 (80.50 90.10)	0.96
Neutrophil count, 10*9/L	11.34 (8.87 14.11)	10.31 (7.80 13.03)	**0.023**
Hematocrit, %	39.3 (33.97 43.20)	39.5 (35.80 42.80)	0.47
Alanine aminotransferase, U/L	26.0 (21.25 37.75)	29.0 (19.80 44.00)	0.66
Aspartate aminotransferase, U/L	35.0 (27.00 67.25)	31.0 (24.00 48.00)	**0.022**
Total cholesterol, mmol/L	3.92 (3.92 3.92)	3.92 (3.92 3.92)	0.65
Urea nitrogen, mmol/L	6.55 (5.62 8.45)	6.60 (5.30 8.20)	0.48
Serum creatinine, umol/L	95.9 (70.4 139.7)	77.3 (61.0 104.5)	**<0.001**
eGFR, ml/min/1.73 m^2^	98.6 (62.6 127.9)	98.6 (69.4 124.8)	0.91
Plasma albumin, g/L	37.8 (35.8 40.5)	38.8 (36.0 41.4)	0.089
Hemoglobin, g/L	133.5 (110.5 147.0)	133.0 (120.0 147.0)	0.44
Low density lipoprotein, mmol/L	2.16 (1.76 2.40)	2.20 (1.73 2.77)	0.55
Lymphocyte percentage, *n* (%)	7.0 (4.82 10.33)	7.5 (5.20 11.20)	0.18
Lymphocyte count, 10*9/L	0.88 (0.63 1.19)	0.91 (0.64 1.25)	0.80
Monocyte percentage, *n* (%)	5.95 (4.73 7.50)	5.70 (4.00 7.70)	0.17
Monocyte count, 10*9/L	0.82 (0.51 1.10)	0.67 (0.45 0.94)	**0.005**
Eosinophil percentage, *n* (%)	0.01 (0.01 0.10)	0.10 (0.01 0.20)	0.27
Eosinophil count, 10*9/L	0.00 (0.00 0.02)	0.01 (0.00 0.02)	0.31
Basophil percentage, *n* (%)	0.20 (0.10 0.20)	0.10 (0.10 0.20)	0.54
Basophil count, 10*9/L	0.02 (0.01 0.03)	0.02 (0.01 0.03)	0.18
Platelet count, 10*9/L	152 (118.0 179.0)	159 (127.0 198.0)	0.10
D-dimer, mg/L	8.88 (5.29 28.15)	5.47 (2.90 13.74)	**<0.001**
Fibrinogen, g/L	2.1 (1.5 2.5)	2.2 (1.7 2.9)	**0.044**
LMR	1.18 (0.86 1.65)	1.38 (0.97 1.97)	**0.004**

AKI, acute kidney injury; CPB, cardiopulmonary bypass; eGFR, estimated Glomerular filtration rate; LMR, lymphocyte to monocyte ratio.

Bold values mean the *p* value <0.05.

^a^ Pearson's Chi-squared test; Wilcoxon rank sum test.

Comparing the Survived group, the deceased group had higher WBC count, lower neutrophil count, higher AST, higher serum creatinine, higher monocyte count, higher D-dimer, lower fibrinogen, and lower LMR (*P* < 0.05). But there was no significant difference in lymphocyte percentage (*P* = 0.18) and lymphocyte count (*P* = 0.80) between the two groups.

### Univariate and multivariate logistic regression analyses of mortality of patient

In summary, we included preoperative and in-surgery confounders to assess the whole cohort; these variables were demographic characteristics, surgery details (prolonged CPB time), and preoperative inflammatory markers. In univariate logistic regression analysis, age, time to onset, prolonged CPB time, serum creatinine, platelet count, D-dimer, fibrinogen, and LMR were associated with 30-day mortality (Table 2).

**Table 2:** Univariate and multivariate logistic regression analysis of deceased and survived groups.

Characteristic	Univariate regression			Multivariate regression		
	OR^a^	95% CI^a^	*P*-value	OR^a^	95% CI^a^	*P*-value
Female	1.264	0.782, 1.993	0.320			
Age	0.979	0.964, 0.995	**0.010**	0.974	0.954, 0.994	**0.013**
Body mass index	1.028	0.977, 1.083	0.300			
Time to onset	2.134	1.106, 4.654	**0.036**	1.633	0.580, 5.300	0.380
Prolonged CPB time	0.425	0.246, 0.700	**<0.001**	0.524	0.281, 0.933	**0.034**
Hematocrit	1.017	0.984, 1.050	0.310			
Alanine aminotransferase	1.000	0.999, 1.002	0.990			
Aspartate aminotransferase	1.000	0.999, 1.001	0.610			
Urea nitrogen	0.986	0.896, 1.111	0.800			
Serum creatinine	0.998	0.997, 0.999	**0.002**	0.998	0.997, 0.999	**0.001**
eGFR	1.003	0.985, 1.022	0.790			
Plasma albumin	1.021	0.973, 1.068	0.380			
Hemoglobin	1.006	0.997, 1.015	0.200			
Low density lipoprotein	1.260	0.765, 2.147	0.380			
Platelet count	1.004	1.000, 1.008	**0.043**	1.001	0.996, 1.006	0.713
D-dimer	0.996	0.992, 0.999	**0.010**	0.996	0.992, 1.000	**0.023**
Fibrinogen	1.277	1.056, 1.577	**0.017**	1.051	0.779, 1.441	0.749
LMR	1.309	1.052, 1.705	**0.030**	1.427	1.070, 2.031	**0.030**

CPB, cardiopulmonary bypass; eGFR, estimated Glomerular filtration rate; LMR, lymphocyte to monocyte ratio.

Bold values mean the *p* value <0.05.

^a^ OR,  odds ratio, CI, confidence interval.

The multivariate logistic regression analysis showed that age (OR = 0.974, 95% CI 0.954–0.994, *P* = 0.013), prolonged CPB time (OR = 0.524, 95% CI 0.281–0.933, *P* = 0.034), serum creatinine (OR = 0.998, 95% CI 0.997–0.999, *P* = 0.001), and D-dimer (OR = 0.996 95% CI 0.992–1.000, *P* = 0.023) were independent risk factors for 30-day mortality (Table 2). It was also found that LMR (OR = 1.427, 95% CI 1.070–2.031, *P* = 0.030) remained significant after adjusting these confounders. Replacing LMR with monocyte count as a sensitivity analysis. The monocyte count was not statistically significant (OR = 0.664, 95% CI 0.314–1.403, *P* = 0.283) after adjusting.

**Figure 2:**
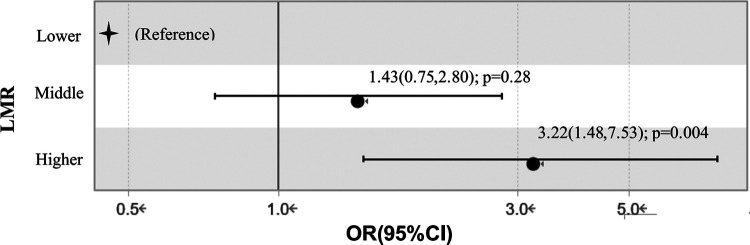
Relationship between different lymphocyte to monocyte ratio levels and in-hospital mortality in multiple factor logistic regression analysis. Adjustment: age, time to onset, CPB time, ventilation time, serum creatinine, platelet, D-dimer, fibrinogen. LMR, lymphocyte to monocyte ratio; OR, odds ratio.

We dichotomized the TAAD cohort into three groups through trisection of LMR concentrations. When the Lower group (<1.072) was set as the reference value, the OR values of the Higher group (>1.689) and Middle group (1.072–1.689) were 3.22 (95% CI: 1.48–7.53; *P* = 0.004) and 1.43 (95% CI: 0.75–2.80; *P* = 0.28) after adjusting for age, time to onset, prolonged CPB time, serum creatinine, platelet, D-dimer and fibrinogen in multiple factor logistic regression analysis, see Figure 2.

## Discussion

To our knowledge, this study has the largest sample cohort that investigates the relevance of LMR on admission with prognosis in patients with TAAD undergoing surgical treatment. The results provided that the decreased LMR is associated with an increased incidence of in-hospital mortality, which identified LMR as an independent risk factor for in-hospital mortality.

TAAD is a life-threatening disease that the ascending aorta is involved. Although the treatment of TAAD has made great progress and the mortality rate has decreased significantly, a significant number of patients still die after receiving surgical treatment (14). Now, there is still a lack of a unified standard to evaluate which TAAD patients are suitable for surgical treatment and which TAAD patients are not. Therefore, it is extremely necessary to identify TAAD patients with a high risk of death during surgery and recommend these patients choose other more optimized treatment options. It is widely known that age, CPB time, ventilation time, serum creatinine and D-dimer are independent risk factors for AD, which are consistent with our results (1, 15–18).

In addition, many studies have also shown inflammation plays important roles in AD (19–22). Medial degeneration is a major histologic feature of AD with massive inflammatory cell infiltration (23). These inflammatory cells promote the apoptosis of smooth muscle cells in the aortic tissue, which leads to AD. Inflammatory cell activity in aortic wall was higher in patients with severe clinical symptoms and progression than in asymptomatic and clinically stable patients (24). All these raise the possibility that the inflammatory indicators may be an important predictor to effectively evaluate the prognosis of AD patients.

Inflammatory cells, including monocytes, lymphocytes and plasma cells, infiltrate the aortic wall through the adventitia, wound tissue or vascular pathways, participate in the destruction of the vascular wall, and thus promote the rupture of the aorta (25, 26). These infiltrating cells are the main source of inflammatory cytokines, which are significantly increased in AD and play multiple roles in regulating lymphocyte activation, endothelial cell proliferation, vascular smooth muscle cell apoptosis and vascular adhesion molecule expression (25). It is undeniable that all of these roles may affect the prognosis of AD.

Lymphocytes are the main inflammatory cells of AD. The occurrence of AD would cause a strong stress reaction in the body, promote the activation of neurohumors, and release inflammatory mediators, which leads to the change in the number of lymphocytes (24). Patients with poor prognosis are often accompanied by significant and persistent lymphopenia. Wu D et al. (27) showed lymphatic infiltration was correlated with aortic dissection rupture, and peripheral blood lymphocyte count was significantly correlated with lymphoid infiltration. He et al. (28) found lymphocytes promote the apoptosis of smooth muscle cells by activating the death-promoting pathways in the aortas of these patients.

Monocytes in the circulatory system accumulate in and under the intima of blood vessels and differentiate into macrophages and start to promote inflammatory response, with the action of some cytokines. The inflammatory activation of monocytes promotes the oxidation of low-density lipoprotein and the oxidative state of endothelial cell activation (28). And high monocyte count at admission is closely related to a poor prognosis of the acute coronary syndrome (28). Monocyte phenotype transformation has become one of the specific therapeutic targets for the prevention and treatment of cardiovascular diseases. In this study, we also found peripheral blood high counts of monocyte are increased in patients of the Deceased group, which may be associated with a more intense inflammatory response.

As an independent factor, LMR, which is associated with lymphocytes and monocytes at the same time, reflects two immune pathways that may be less affected by confounding conditions and may be more predictive than monocytes or lymphocytes alone in assessing the prognosis of diseases. LMR had been used to predict adverse events of various cardiovascular diseases (8, 29, 30). However, the potential application value of LMR to provide prognostic information for patients with TAAD remains unclear. In this study, we found lower LMR was associated with higher in-hospital mortality after surgical treatment for TAAD. That maybe due to higher monocytes and associated with a more severe inflammatory response, based on the evidence of our study.

## Limitation

Some limitations should be detailed in this study. First, this is a retrospective study confounding factors may have affected the results, although we avoid these to the greatest degree with a multivariate analysis. Second, this study was a single-center study, the predictive value of LMR needs a larger sample size and the data of multicenter trials to further confirm. Third, LMR was not detected dynamically. It is not clear whether a continuous measurement of the dynamic change of LMR is more valuable to evaluate the relationship between LMR and the prognosis of TAAD. Fourthly, the multiple impacts of complex conditions on LMR cannot be ruled out completely.

## Conclusion

LMR has been proven to be an independent factor in patients with TAAD receiving surgical treatment, and lower LMR levels at hospital admission are associated with higher in-hospital mortality. LMR can be used as an important factor to stratify the prognosis risk of TAAD patients, identify high-risk patients, guide treatment strategies, and reduce the risk of postoperative death.

## Data Availability

The raw data supporting the conclusions of this article will be made available by the authors, without undue reservation.
